# Lateral abdominal wall hematoma as a rare complication after carotid artery stenting: a case report

**DOI:** 10.1186/1749-7922-4-39

**Published:** 2009-11-12

**Authors:** Naoto Fukunaga, Shizuo Ikeyama, Jyunichiro Satomi, Koichi Satoh

**Affiliations:** 1 Department of Neurosurgery, Tokushima Red Cross Hospital, 103 Irinoguchi, Komatsushima-cho, Komatsushima-shi, Tokushima 773-8502, Japan; 2 Department of Radiology, Tokushima Red Cross Hospital, 103 Irinoguchi, Komatsushima-cho, Komatsushima-shi, Tokushima 773-8502, Japan; 3 Department of Neurosurgery, Tokushima University Hospital, 50-1-2 Kuramoto-cho, Tokushima-shi, Tokushima 770-8503, Japan; 4 Current address: Department of Cardiovascular surgery, Kobe City Medical Center General Hospital, 4-6 Minatojimanakamachi, Chuo-ku, Kobe-shi, Hyogo 650-0046, Japan

## Abstract

Abdominal wall hematoma is a rare and life-threatening complication after carotid artery stenting (CAS), but it can occur when activated clotting time is prolonged. We report a right lateral abdominal wall hematoma caused by rupture of the superficial circumflex iliac artery after CAS in a 72-year-old man with severe stenosis of the origin of the right internal carotid artery. We performed CAS for the targeted lesion while activated clotting time exceeded 300 seconds. After 2 hours, he complained of right lateral abdominal pain. Abdominal computed tomography revealed an extensive hematoma in the right lateral abdominal wall. Activated clotting time was 180 seconds at this point. Seven hours later, he developed hypotension and hemoglobin level dropped to 11.3 g/dl. Subsequent computed tomography showed enlargement of the hematoma. Emergent selective angiography of the external iliac artery revealed active bleeding from the right superficial circumflex iliac artery. Transcatheter arterial embolization with Gelfoam and microcoils was performed successfully. With more CAS procedures being performed, it is important for endovascular surgeons and radiologists to consider the possibility of abdominal wall hematoma in this situation.

## Introduction

Lateral abdominal wall hematoma is a rare condition that can give rise to an acute abdomen [1]. Predisposing factors include anticoagulant therapy [1-3]. With the increase in carotid artery stenting in patients in whom activated clotting time is prolonged for prevention of cerebral infarction, we must be aware of the possibility of abdominal wall hematoma. Moreover, accurate diagnosis allows us to avoid unnecessary surgical intervention. We report a right lateral abdominal wall hematoma caused by rupture of the superficial circumflex iliac artery after carotid artery stenting. The hematoma was treated by transcatheter arterial embolization.

## Case presentation

**Figure 1:**
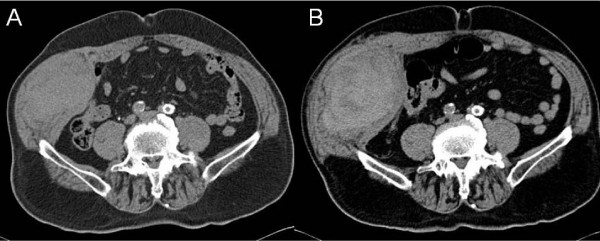
**(A) Abdominal computed tomography (CT) shows the extensive hematoma in the right lateral abdominal wall 2 hours after carotid artery stenting**. (B) Abdominal CT clearly shows enlargement of the hematoma 7 hours after the first CT.

**Figure 2:**
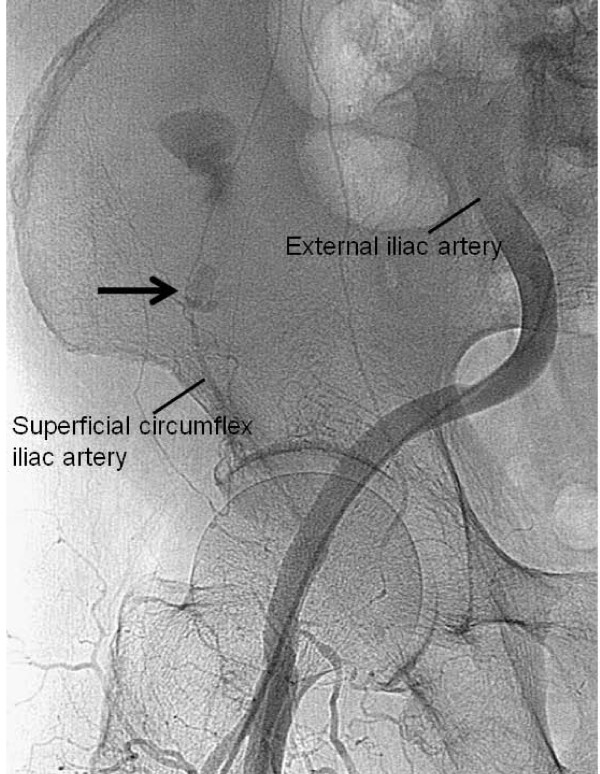
**Emergent selective angiography of the external iliac artery shows active bleeding from the right superficial circumflex iliac artery (arrow)**. Transcatheter arterial embolization with Gelfoam and microcoils was performed successfully.

A 72-year-old man with no neurological symptoms was admitted to our hospital because of severe stenosis of the origin of the right internal carotid artery. We performed carotid artery stenting for the targeted lesion with an activated clotting time of more than 300 seconds, and good patency was obtained. Postoperative magnetic resonance imaging showed no evidence of cerebral infarction. After 2 hours, he complained of right lateral abdominal pain. Abdominal computed tomography revealed an extensive hematoma in the right lateral abdominal wall; at this stage, activated clotting time was 180 seconds (Fig. 1A). Because he was alert and hemodynamically stable at that time, we opted for watchful waiting. After 7 hours the patients developed nausea, and had a regular pulse of 140 beats per minute and a systolic blood pressure of 80 mmHg. Hemoglobin level dropped from 13.9 to 11.3 g/dl. Subsequent computed tomography showed enlargement of the hematoma (Fig. 1B). Emergent selective angiography of the external iliac artery revealed active bleeding from the right superficial circumflex iliac artery (Fig. 2). After red blood cell transfusions, transcatheter arterial embolization with Gelfoam and microcoils was performed successfully. The postoperative course was uneventful and he was discharged on the 14th day. To date, no recurrence of the right lateral abdominal wall hematoma has been recognized.

## Conclusion

Spontaneous rectus sheath hematoma is a rarely diagnosed condition [2] with rupture of the inferior epigastric artery being a well-known cause [3]. An expanding abdominal wall hematoma is also a rare cause of acute abdomen [1]. Intravascular procedures on targeted vessels such as the iliac artery [1,4,5] and subcostal artery [6] have been reported as a cause of abdominal wall hematoma. However, the literature contains no reports of abdominal wall hematoma caused by rupture of the superficial circumflex iliac artery after carotid artery stenting (CAS). Although there is one report of spontaneous rectus sheath hematoma as a complication of CAS, that was caused by rupture of the deep circumflex iliac artery [5]. To the best of our knowledge, this is the first report of lateral abdominal wall hematoma caused by rupture of the superficial circumflex iliac artery after CAS.

Lateral abdominal wall hematoma can occur as a result of non-traumatic injury such as iatrogenic injury to vessels or abdominal muscles, in presence of predisposing factors [6]. These include overcontraction or overstretching of muscles from coughing, sneezing, twisting or vomiting, weakness of the vessel wall or decrease in muscular resistance as a result of hypertension, arteriosclerosis, advanced age, obesity, pregnancy or previous surgery, and bleeding tendency or use of anticoagulants [1-3]. In the present case, we performed CAS while activated clotting time remained prolonged for prevention of cerebral infarction, and the catheter injured the superficial circumflex iliac artery. This induced lateral abdominal wall hematoma, which resulted in shock.

Accurate diagnosis of acute abdominal diseases can help surgeons avoid unnecessary operations. Because of its rarity, abdominal wall hematoma has been mistaken for common acute abdominal condition including appendicitis, incarcerated inguinal hernia, acute cholecystitis, acute aortic dissection, complications of pregnancy and ovarian torsion [7]. Ultrasonography and computed tomography (CT) are useful modalities for differential diagnosis and can reduce unnecessary surgery for abdominal rectus sheath hematoma [8]. In addition to contrast-enhanced CT can detect and evaluate active bleeding from the rupture site [6].

Conservative treatment including bed rest and analgesics is appropriate for most patients [2]. Surgery is reserved for rupture into free peritoneum, infection and progression of the hematoma [2]. Recently, reports have demonstrated that transcatheter arterial embolization is an effective and less invasive method to control the active bleeding, allowing surgery to be avoided [1,6].

Abdominal wall hematoma is a rare and life-threatening complication after CAS, but is possible when activated clotting time is prolonged. If suggestive symptoms develop, clinicians have the opportunity to investigate the causes with CT or ultrasonography. If this is not performed, active bleeding might continue and endanger the patient's life. With the increase in CAS procedures, it is important for endovascular surgeons and radiologists to take into consideration the possibility of abdominal wall hematoma in this situation.

## Consent

Written informed consent was obtained from the patient for publication of this case report and any accompanying images. A copy of the written consent is available for review by the Editor-in-Chief of this journal.

## Competing interests

The authors declare that they have no competing interests.

## Authors' contributions

NF wrote this manuscript and revised it. SI, JS and KS performed the operation and recommended me to write this case and advised me to revise it. All authors read and approved the final manuscript.
